# Three-year outcomes of post-acute sequelae of COVID-19

**DOI:** 10.1038/s41591-024-02987-8

**Published:** 2024-05-30

**Authors:** Miao Cai, Yan Xie, Eric J. Topol, Ziyad Al-Aly

**Affiliations:** 1 Clinical Epidemiology Center, Research and Development Service, VA St. Louis Health Care System, St. Louis, MO USA; 2 Veterans Research and Education Foundation of St. Louis, St. Louis, MO USA; 3 Division of Pharmacoepidemiology, Clinical Epidemiology Center, Research and Development Service, VA St. Louis Health Care System, St. Louis, MO USA; 4 Scripps Research, La Jolla, CA USA; 5 Department of Medicine, Washington University School of Medicine, St. Louis, MO USA; 6 Nephrology Section, Medicine Service, VA St. Louis Health Care System, St. Louis, MO USA; 7 Institute for Public Health, Washington University in St. Louis, St. Louis, MO USA

**Keywords:** Viral infection, SARS-CoV-2

## Abstract

Severe acute respiratory syndrome coronavirus 2 (SARS-CoV-2) infection causes post-acute sequelae of coronavirus disease 2019 (COVID-19) (PASC) in many organ systems. Risks of these sequelae have been characterized up to 2 years after infection, but longer-term follow-up is limited. Here we built a cohort of 135,161 people with SARS-CoV-2 infection and 5,206,835 controls from the US Department of Veterans Affairs who were followed for 3 years to estimate risks of death and PASC. Among non-hospitalized individuals, the increased risk of death was no longer present after the first year of infection, and risk of incident PASC declined over the 3 years but still contributed 9.6 (95% confidence interval (CI): 0.4–18.7) disability-adjusted life years (DALYs) per 1,000 persons in the third year. Among hospitalized individuals, risk of death declined but remained significantly elevated in the third year after infection (incidence rate ratio: 1.29 (95% CI: 1.19–1.40)). Risk of incident PASC declined over the 3 years, but substantial residual risk remained in the third year, leading to 90.0 (95% CI: 55.2–124.8) DALYs per 1,000 persons. Altogether, our findings show reduction of risks over time, but the burden of mortality and health loss remains in the third year among hospitalized individuals.

## Abstract (web summary)

Analyses from the US Department of Veterans Affairs databases reported residual elevated risk and health burden of long COVID at 3 years in hospitalized individuals after SARS-CoV-2 infection.

## Main

Severe acute respiratory syndrome coronavirus 2 (SARS-CoV-2) infection leads to long-term health effects in nearly every organ system, collectively referred to by the patient-coined term Long Covid^1–14^. Studies following infected individuals for 1 year and 2 years described risk trajectories for many conditions^1,3–13,15–19^. Risks for some conditions abate after the first year after infection, but risks for many conditions persist at 2 years after initial infection, especially among individuals who were hospitalized for coronavirus disease 2019 (COVID-19) during the acute phase of illness^20^. About 25% of the burden of the 2-year cumulative burden of disability and disease due to SARS-CoV-2 emanates from the second year after initial infection^20^. However, studies with longer follow-up times are limited^21^. It is unclear whether and to what extent risks remain in the third year after infection and whether new latent risks (that have not yet been observed) become apparent in the third year after infection.

Accordingly, we undertook a comprehensive assessment of the risks and burdens of post-acute sequelae of COVID-19 (PASC) across care settings of the acute infection—both non-hospitalized and hospitalized individuals—in the 3 years after infection. Addressing this knowledge gap is important to deepen understanding of the post-acute and long-term health trajectories of people who had SARS-CoV-2 infection and will inform care of people with these conditions.

In this work, we used the US Department of Veterans Affairs (VA) national health care databases to build a cohort of 135,161 US veterans who survived the first 30 d of COVID-19 and a control of 5,206,835 users of the VA healthcare system with no evidence of SARS-CoV-2 infection. To ensure 3-year follow-up, these cohorts were enrolled between March and December 2020, an era that pre-dated the availability of COVID-19 vaccines and antivirals and when the ancestral SARS-CoV-2 virus predominated. These cohorts were followed longitudinally for 3 years to estimate the risks of death and incident sequelae of SARS-CoV-2 throughout the 3-year follow-up and cumulatively at 3 years in mutually exclusive groups according to care setting of the acute phase of the disease (in non-hospitalized and hospitalized).

## Results

**Extended Data Fig. 1:**
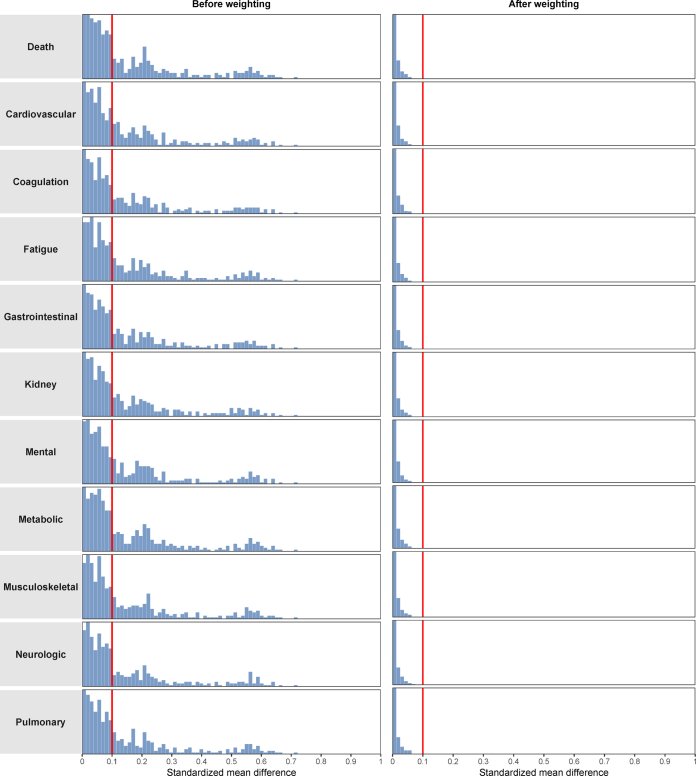
Standardized mean differences between non-hospitalized, hospitalized, and control groups before and after weighting in analyses of post-acute sequelae (PASC) and sequelae by organ system. Plots show the standardized mean differences before weighting (left) and after weighting (right). Each row represents a sub-cohort used in analyzing the risks of PASC by organ system that was free of history of the respective outcomes at baseline. All selected variates had a standardized mean difference < 0.1 after weighting.

There were 114,864 participants (13,810 (12.0%) females and 101,054 (88.0%) males) in the non-hospitalized COVID-19 group and 20,297 participants in the hospitalized COVID-19 group (1,177 (5.8%) females and 19,120 (94.2%) males), and there were 5,206,835 participants in the control group without infection (503,509 (9.7%) females and 4,703,326 (90.3%) males). All participants had a full 3 years of follow-up, totaling 344,592, 60,891 and 15,620,505 person-years of follow-up in the non-hospitalized COVID-19, hospitalized COVID-19 and control groups, respectively. Altogether, this corresponded to 16,025,988 person-years of follow-up. Demographic, health characteristics and standardized mean differences of the non-hospitalized COVID-19, hospitalized COVID-19 and control groups before and after inverse probability weighting for baseline covariates are presented in Supplementary Tables 1 and 2 and Extended Data Fig. 1.

We examined risks and burdens of death and a set of pre-specified PASC as well as sequelae aggregated by organ system and aggregated as an overall outcome of PASC by care setting during the acute phase of SARS-CoV-2 infection (non-hospitalized (*n* = 114,864) and hospitalized (*n* = 20,297) groups) in the first, second and third year after SARS-CoV-2 infection.

### Risks in non-hospitalized participants

**Fig. 1:**
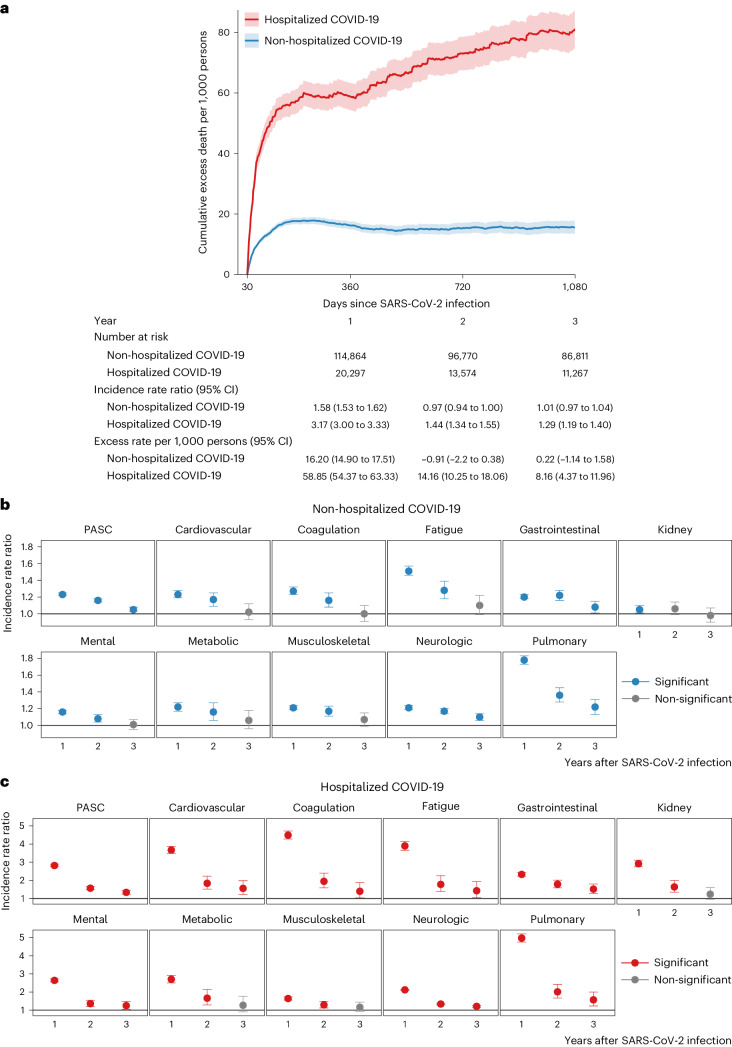
Cumulative excess death rate and incidence rate ratio of PASC in COVID-19 groups by care setting of the acute phase. **a**, The solid lines at the center of shaded bands were adjusted cumulative excess death rate per 1,000 persons in non-hospitalized COVID-19 (*n* = 114,864) and hospitalized COVID-19 (*n* = 20,297) groups compared to the control group without infection (*n* = 5,206,835), and the shaded bands present the 95% CIs for cumulative excess rates. The number at risk, weighted IRR and weighted excess rate per 1,000 persons in the COVID-19 groups by care setting of the acute phase compared to the control group without infection are also presented in the lower panel. **b**, IRR of overall PASC and by organ system in non-hospitalized COVID-19 group (*n* = 114,864) compared to the control group without infection (*n* = 5,206,835). The dots at the center of error bars in both panels represent the adjusted IRRs estimated using the number of post-acute sequelae, and the error bars correspond to the 95% CIs. **c**, IRR of overall PASC and by organ system in hospitalized COVID-19 group (*n* = 20,297) compared to the control group without infection (*n* = 5,206,835). Outcomes are ordered from top to bottom by largest cumulative number of post-acute sequelae at 3 years after infection in the non-hospitalized COVID-19 group. The dots at the center of error bars in both panels represent the adjusted IRRs estimated using the number of post-acute sequelae, and the error bars correspond to the 95% CIs.

Compared to the control group without infection, people with COVID-19 who were not hospitalized during the acute phase of the disease were at an increased risk of death (incidence rate ratio (IRR): 1.58, 95% confidence interval (CI): 1.53–1.62; excess burden per 1,000 persons: 16.20, 95% CI: 14.90–17.51; Fig. 1a) during the first year after SARS-CoV-2 infection but not in the second year (IRR: 0.97, 95% CI: 0.94–1.00; excess burden per 1,000 persons: −0.91, 95% CI: −2.20 to 0.38) and third year (IRR: 1.01, 95% CI: 0.97–1.04; excess burden per 1,000 persons: 0.22, 95% CI: −1.14 to 1.58) after the infection.

**Fig. 2:**
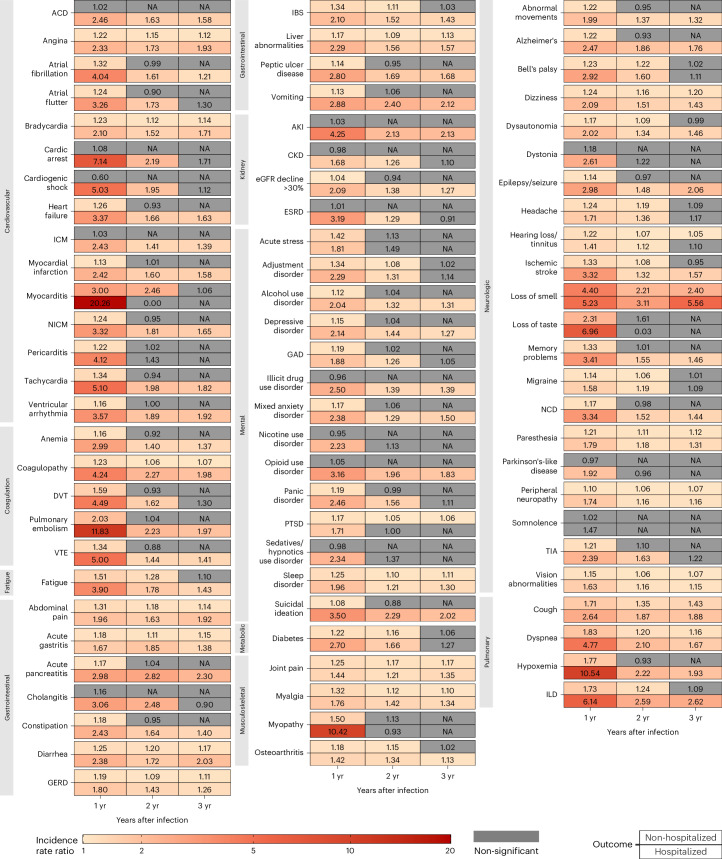
IRR of PASC up to 3 years after SARS-CoV-2 infection by care setting of the acute phase. Heatmaps include non-hospitalized COVID-19 (*n* = 114,864; top rows) and hospitalized COVID-19 (*n* = 20,297; bottom rows) groups. IRRs were estimated in comparison to a control group without infection (*n* = 5,206,835). ACD, acute coronary disease; AKI, acute kidney injury; CKD, chronic kidney disease; DVT, deep vein thrombosis; ESRD, end-stage renal disease; GAD, general anxiety disorder; GERD, gastroesophageal reflux disease; IBS, irritable bowel syndrome; ICM, ischemic cardiomyopathy; ILD, interstitial lung disease; NA, not applicable; NCD, neurocognitive decline; NICM, non-ischemic cardiomyopathy; PTSD, post-traumatic stress disorder; TIA, transient ischemic attack; VTE, venous thromboembolism. If potential risk horizon (non-significant (NS) cell with a numeric IRR estimate) for an outcome was reached in a previous period, the IRRs for that outcome in all subsequent periods will not be estimated and are indicated by gray cells with NAs (not applicable) inside. yr, year.

**Extended Data Fig. 2:**
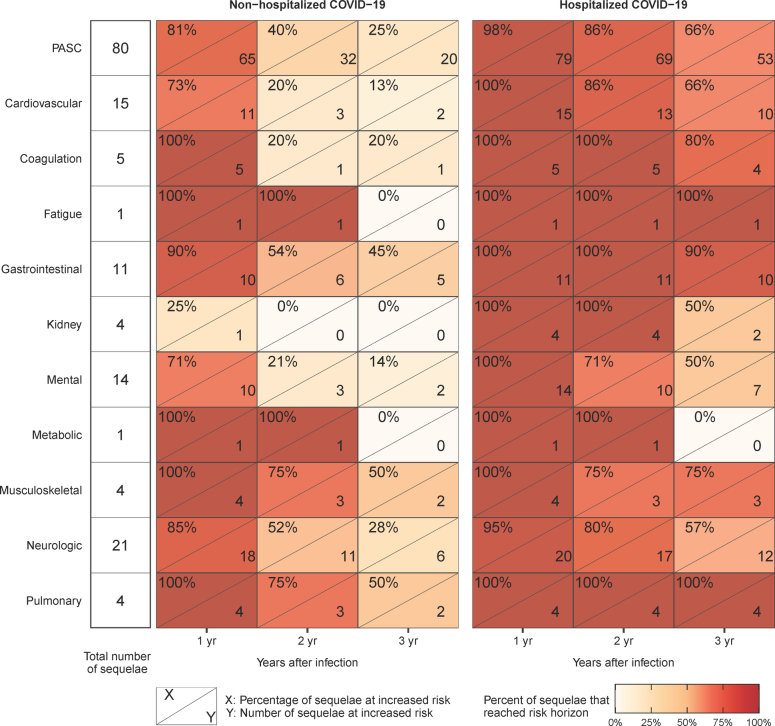
Summary of risk of post-acute sequelae of COVID-19 (PASC) by years of follow-up and care setting of the acute phase. The figure presents the summaries of PASC by organ system for non-hospitalized COVID-19, hospitalized COVID-19 in each year of follow-up after SARS-CoV-2 infection. The number in the leftmost column with white background denotes the total number of sequelae examined in each organ system. Within each cell, the top left percent represent the percentage of number of sequelae at increased risk in non-hospitalized/hospitalized COVID-19 compared to the control, while the bottom right number is the number of sequelae in the organ system in that year.

The risks on the relative scale (IRRs) of PASC were 1.23 (95% CI: 1.22–1.25), 1.16 (1.14–1.18) and 1.05 (1.03–1.08) in the first, second and third year, respectively (Fig. 1b and Supplementary Table 3). The risks of individual outcomes in years 1, 2 and 3 are presented in Fig. 2, Supplementary Table 4 and Extended Data Fig. 2.

**Fig. 3:**
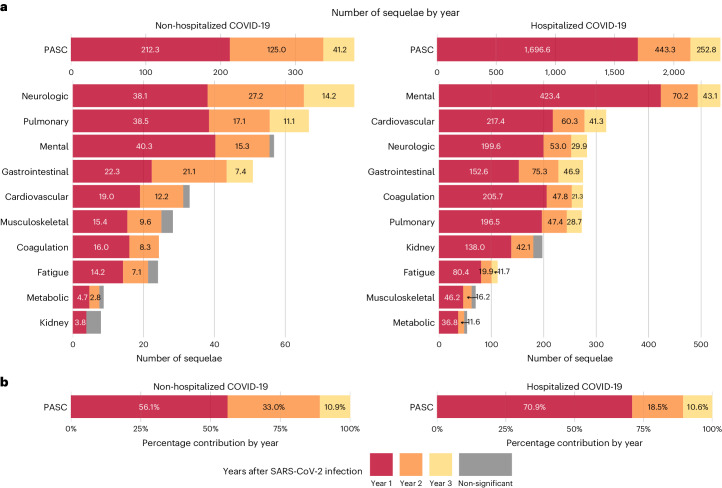
Number of sequelae due to SARS-CoV-2 infection for overall PASC and by organ system and relative percentage for overall PASC in the first, second and third year after SARS-CoV-2 infection by care setting of the acute phase compared to the control group without infection. **a**, Number of post-acute sequelae overall and by organ system per 1,000 persons in the first, second and third year after SARS-CoV-2 infection by care setting of acute phase. **b**, Relative percentage of number of post-acute sequelae overall in the first, second and third year after SARS-CoV-2 infection by care setting of acute phase. Number of post-acute sequelae for COVID-19 not significantly different from the control group without infection in a year is marked by gray bars. The left column represents numbers of post-acute sequelae for the non-hospitalized COVID-19 group (*n* = 114,864), and the right column represents the numbers for the hospitalized COVID-19 group (*n* = 20,297), compared to the control group without infection (*n* = 5,206,835). Outcomes are ordered from top to bottom by cumulative number of post-acute sequelae at 3 years after infection.

The cumulative number of post-acute sequelae over the 3 years was 378.7 (95% CI: 356.6–401.1) per 1,000 persons, including 212.3 (197.5–227.0), 125.0 (107.2–142.7) and 41.2 (20.2–62.3) in the first, second and third year, respectively, which corresponded to 56.1% (52.2–60.0%), 33.0% (28.3–37.7%) and 10.9% (5.3–16.5%) of the total 3-year cumulative burden of PASC, respectively (Fig. 3 and Supplementary Table 3).

**Fig. 4:**
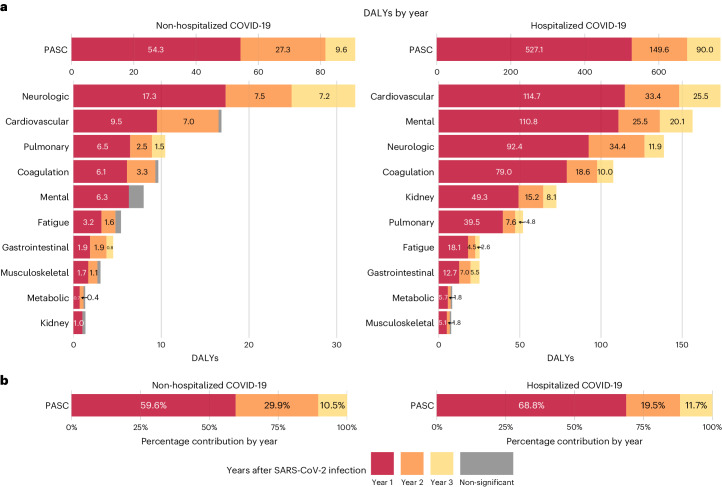
DALYs due to SARS-CoV-2 infection for overall PASC and by organ system and relative percentage for overall PASC in the first, second and third year after SARS-CoV-2 infection by care setting of the acute phase compared to the control group without infection. **a**, DALYs of overall PASC and by organ system per 1,000 persons in the first, second and third year after SARS-CoV-2 infection by care setting of acute phase. **b**, Relative percentage of DALYs of overall PASC in the first, second and third year after SARS-CoV-2 infection by care setting of acute phase. DALYs for COVID-19 not significantly different from the control group without infection in a year are marked by gray bars. The left panels of **a** and **b** represent the DALYs of post-acute sequelae for the non-hospitalized COVID-19 group (*n* = 114,864), and the right panels of **a** and **b** represent the number for the hospitalized COVID-19 group (*n* = 20,297), compared to the control group without infection (*n* = 5,206,835). Outcomes are ordered from top to bottom by cumulative DALYs of post-acute sequelae at 3 years after infection.

The 3-year cumulative burden of DALYs due to PASC was 91.2 (95% CI: 81.6–101.0) per 1,000 persons, including 54.3 (47.9–60.7), 27.3 (19.5–35.0) and 9.6 (0.4–18.7) in the first, second and third year, respectively, which corresponded to 59.6% (52.5–66.6%), 29.9% (21.4–38.4%) and 10.5% (0.4–20.6%) of the total 3-year cumulative DALYs, respectively (Fig. 4 and Supplementary Table 3).

**Fig. 5:**
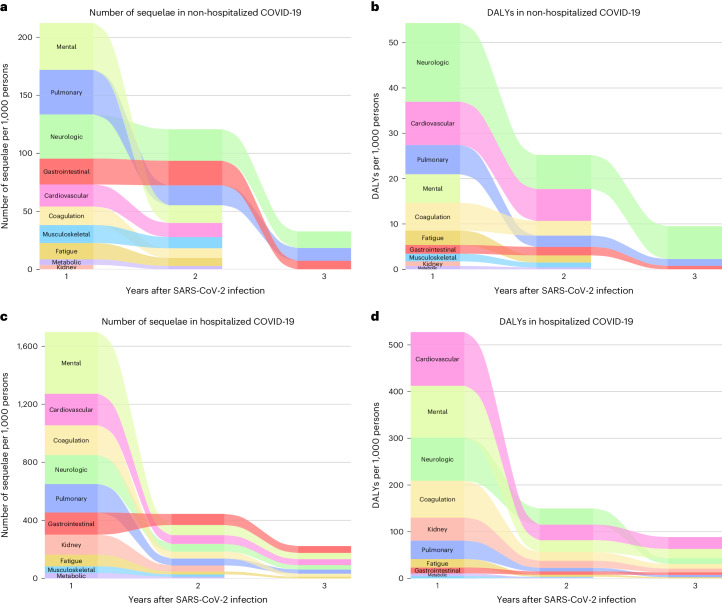
Sanky plot of changes of number of sequelae or DALYs of PASC by organ system in the first, second and third year after SARS-CoV-2 infection by care setting of the acute phase compared to the control group without infection. **a**, Changes of number of post-acute sequelae by organ system in 3 years after SARS-CoV-2 infection in the non-hospitalized COVID-19 group (*n* = 114,864). **b**, Changes of DALYs of post-acute sequelae by organ system in 3 years after SARS-CoV-2 infection in the non-hospitalized COVID-19 group (*n* = 114,864). **c**, Changes of number of post-acute sequelae by organ system in 3 years after SARS-CoV-2 infection in the hospitalized COVID-19 group (*n* = 20,297). **d**, Changes of DALYs of post-acute sequelae by organ system in 3 years after SARS-CoV-2 infection in the hospitalized COVID-19 group (*n* = 20,297). The height of each box represents the number of sequelae or DALYs in COVID-19 groups that are significantly different from the control group without infection in each year after SARS-CoV-2 infection. Outcomes are ordered from top to bottom by number/DALYs of post-acute sequelae per 1,000 persons in each year after SARS-CoV-2 infection in the COVID-19 group.

In the analyses for numbers of post-acute sequelae by organ system, the risks and burdens for each organ system in years 1, 2 and 3 for non-hospitalized group are provided in Figs. 1b and 3 and Supplementary Table 3. Compared to the control group without infection, there was increased risk for post-acute sequelae in all 10 organ systems in the first year; nine organ systems (all except kidney disorders) exhibited increased risks in the second year; and three organ systems exhibited increased risks in the third year, including neurologic, pulmonary and gastrointestinal disorders—contributing 14.2 (95% CI: 8.4–20.0), 11.1 (6.3–15.8) and 7.4 (0.8–13.9) sequelae per 1,000 persons in the third year, respectively. This corresponded to 7.2 (1.0–13.4), 1.5 (0.6–2.4) and 0.8 (0.02–1.5) DALYs in the third year for neurologic, pulmonary and gastrointestinal disorders, respectively (Fig. 4 and Supplementary Table 3). A Sankey plot showing the relative ranking of number of sequelae and DALYs by organ system in years 1, 2 and 3 is shown in Fig. 5a,b.

**Extended Data Fig. 3:**
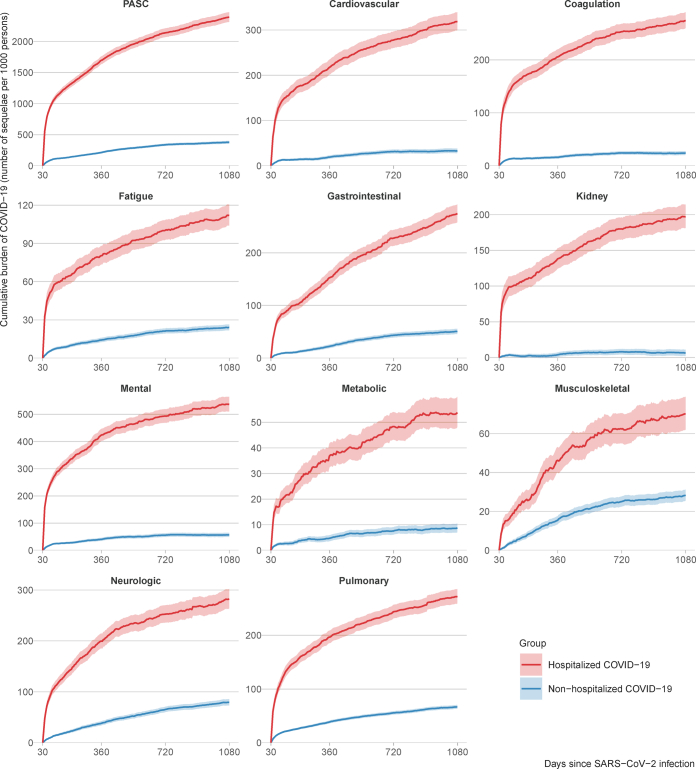
Cumulative number of post-acute sequelae (PASC) overall and by organ system and by care setting of the acute phase. The solid lines at the centre of shaded bands were adjusted cumulative excess number of PASC per 1000 persons in non-hospitalized (n = 114,864) and hospitalized COVID-19 (n = 20,297) groups compared to the control group without infection (n = 5,206,835), and the shaded bands present the 95% confidence intervals for cumulative rates.

**Extended Data Fig. 4:**
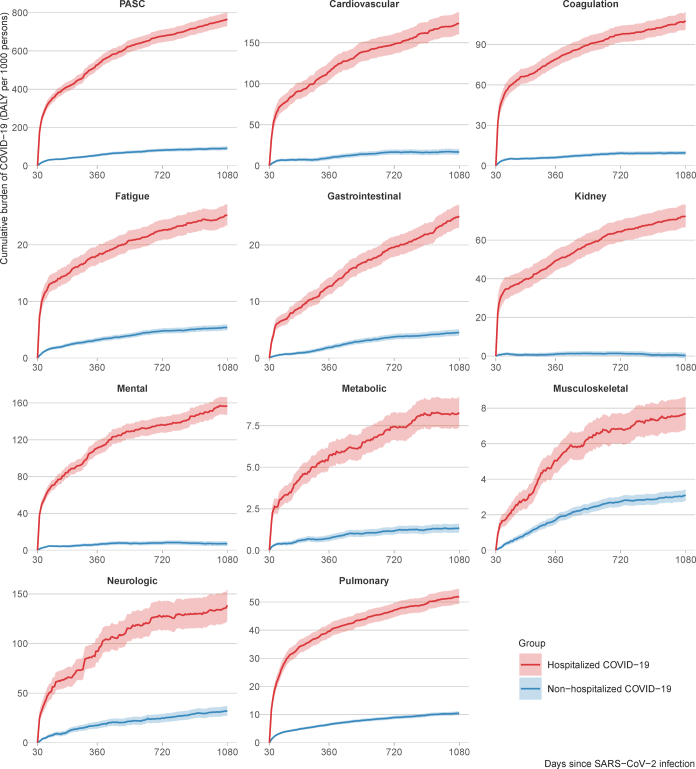
Cumulative disability-adjusted life years (DALYs) of post-acute sequelae (PASC) overall and by organ system and by care setting of the acute phase. The solid lines at the centre of shaded bands were adjusted cumulative excess DALYs of PASC per 1000 persons in non-hospitalized (n = 114,864) and hospitalized COVID-19 (n = 20,297) groups compared to the control group without infection (n = 5,206,835), and the shaded bands present the 95% confidence intervals for cumulative rates.

The 3-year cumulative number of sequelae and DALYs for each organ system are provided in Figs. 3 and 4, Supplementary Table 3 and Extended Data Figs. 3 and 4. Considering DALYs (Fig. 4), and in descending order, the top five organ systems were neurologic, cardiovascular, pulmonary, coagulation and hematologic and mental disorders. The 3-year cumulative number of sequelae per 1,000 persons was 79.5 (73.4–85.7) for neurologic, 33.0 (28.0–38.3) for cardiovascular, 66.7 (63.1–70.4) for pulmonary, 24.1 (20.5–27.9) for coagulation and hematologic and 56.9 (49.2–64.7) for mental disorders. The associated DALYs per 1,000 persons were 32.2 (27.5–37.1) for neurologic, 16.9 (13.4–20.5) for cardiovascular, 10.5 (9.8–11.1) for pulmonary, 9.7 (8.3–11.2) for coagulation and hematologic and 7.2 (4.7–9.8) for mental disorders.

### Risks in hospitalized patients

Compared to the control group without infection, people with COVID-19 who were hospitalized during the acute phase of the disease were at an increased risk of death during the first year (IRR: 3.17, 95% CI: 3.00–3.33; excess burden per 1,000 persons: 58.85, 95% CI: 54.37–63.33; Fig. 1a), during the second year (IRR: 1.44, 95% CI: 1.34–1.55; excess burden per 1,000 persons: 14.16, 95% CI: 10.25–18.06) and during the third year (IRR: 1.29, 95% CI: 1.19–1.40; excess burden per 1,000 persons: 8.16, 95% CI: 4.37–11.96) after SARS-CoV-2 infection.

The risks on the relative scale (IRRs) for post-acute sequelae were 2.82 (95% CI: 2.76–2.89), 1.57 (1.49–1.66) and 1.34 (1.24–1.45) in the first, second and third year, respectively (Fig. 1c and Supplementary Table 5). The risks of individual outcomes in years 1, 2 and 3 are presented in Fig. 2, Supplementary Table 4 and Extended Data Fig. 2.

The cumulative number of post-acute sequelae over the 3 years was 2,391.7 (95% CI: 2,316.0–2,472.3) per 1,000 persons, including 1,696.6 (1,636.6–1,756.6), 443.3 (375.1–511.6) and 252.8 (176.9–328.7) in the first, second and third year, respectively, which corresponded to 70.9% (68.4–73.4%), 18.5% (15.7–21.4%) and 10.6% (7.4–13.7%) of the total 3-year cumulative burden of PASC, respectively (Fig. 3 and Supplementary Table 5).

The 3-year cumulative burden of DALYs due to PASC was 766.2 (95% CI: 731.7–803.3) per 1,000 persons, including 527.1 (499.5– 554.7), 149.6 (118.1–181.0) and 90.0 (55.2–124.8) in the first, second and third year, respectively, which corresponded to 68.8% (65.2–72.4%), 19.5% (15.4–23.6%) and 11.7% (7.2–16.3%) of the total 3-year cumulative DALYs, respectively (Fig. 4 and Supplementary Table 5).

In the analyses for the numbers of post-acute sequelae by organ system, the risks and burdens for each organ system in years 1, 2 and 3 for the hospitalized group are provided in Figs. 1c and 3 and Supplementary Table 5. Compared to the control group without infection, there was increased risk of post-acute sequelae in all 10 organ systems in the first and second year, and seven organ systems (except kidney, metabolic and musculoskeletal) exhibited increased risks in the third year—contributing 41.3 (14.1–68.4) sequelae for cardiovascular, 43.1 (6.2–80.1) for mental, 29.9 (19.3–40.6) for neurologic, 21.3 (0.3–42.2) for coagulation and hematologic, 28.7 (10.1–47.3) for pulmonary, 11.7 (0.2–23.2) for fatigue and 46.9 (24.1–69.8) for gastrointestinal disorders per 1,000 persons in the third year, respectively. This corresponded to 25.5 (7.4–43.6) DALYs for cardiovascular, 20.1 (7.3–33.0) for mental, 11.9 (0.1–23.8) for neurologic, 10.0 (1.1–18.8) for coagulation and hematologic, 4.8 (1.2–8.5) for pulmonary, 2.6 (0.1–5.2) for fatigue and 5.5 (2.8–8.2) for gastrointestinal disorders per 1,000 persons in the third year, respectively (Fig. 4 and Supplementary Table 5). A Sankey plot showing the relative ranking of number of sequelae and DALYs by organ system in years 1, 2 and 3 is shown in Fig. 5c,d.

The 3-year cumulative number of sequelae and DALYs for each organ system are provided in Figs. 3 and 4, Supplementary Table 5 and Extended Data Figs. 3 and 4. Considering DALYs (Fig. 4), and in descending order, the top five organ systems were cardiovascular, mental, neurologic, coagulation and hematologic and kidney disorders. The 3-year cumulative number of sequelae per 1,000 persons was 318.8 (299.5–339.7) for cardiovascular, 536.4 (510.1–564.6) for mental, 282.2 (263.6–302.2) for neurologic, 274.5 (259.7–290.5) for coagulation and hematologic and 197.0 (180.9–214.7) for kidney disorders. The associated DALYs per 1,000 persons were 173.5 (160.5–187.5) for cardiovascular, 156.3 (147.1–166.2) for mental, 138.5 (123.7–155.0) for neurologic, 107.5 (101.3–114.2) for coagulation and hematologic and 72.4 (66.7–78.7) for kidney disorders.

### Risk in hospitalized versus non-hospitalized participants

**Fig. 6:**
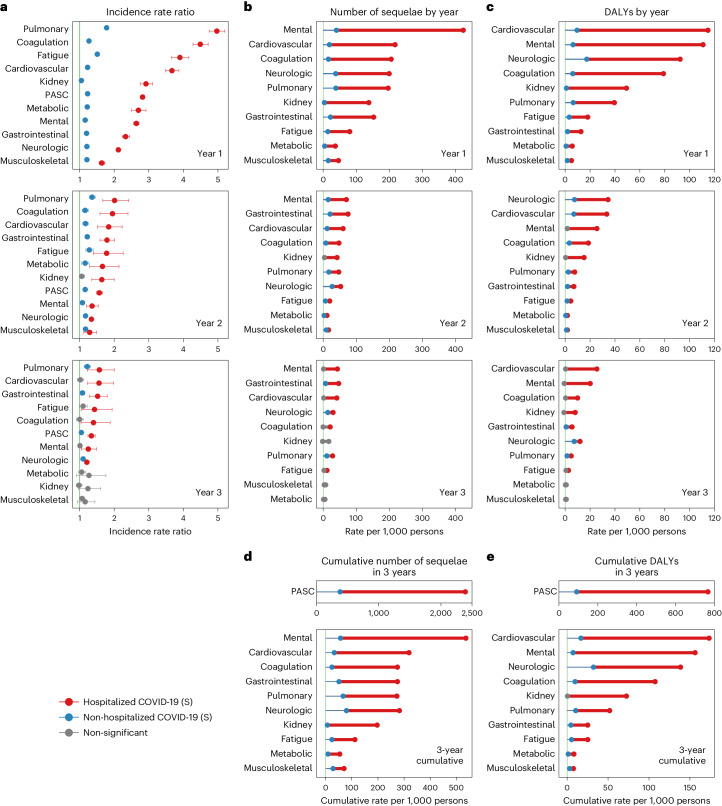
Comparison of overall PASC and at organ system level between hospitalized and non-hospitalized COVID-19 groups over 3 years of follow-up. **a**, IRRs (95% CIs) of number of sequelae for hospitalized (*n* = 114,864) and non-hospitalized (*n* = 20,297) COVID-19 groups by year. IRRs were estimated in comparison to a control group without infection (*n* = 5,206,835). The dots at the center of error bars represent the adjusted IRRs estimated using the number of post-acute sequelae, and the error bars correspond to the 95% CIs. **b**, Number of sequelae per 1,000 persons due to SARS-CoV-2 infection in hospitalized and non-hospitalized COVID-19 groups by year. **c**, DALYs per 1,000 persons due to SARS-CoV-2 infection in hospitalized and non-hospitalized COVID-19 groups by year. **d**, Cumulative number of sequelae per 1,000 persons due to SARS-CoV-2 infection for hospitalized and non-hospitalized COVID-19 groups at 3 years. **e**, Cumulative DALYs per 1,000 persons due to SARS-CoV-2 infection for hospitalized and non-hospitalized COVID-19 groups at 3 years. **a** is ordered by the IRRs among the hospitalized COVID-19 groups in each year. The red and blue dots show the IRRs in hospitalized and non-hospitalized COVID-19 groups significantly larger than 1, and the error bars are the associated CIs. In **b**, **c**, **d** and **e**, the red dots represent the absolute rates in hospitalized COVID-19 groups significantly higher than those in the control group without infection; the blue dots represent the absolute rates in non-hospitalized COVID-19 groups significantly higher than the control group without infection; and the gray dots represent the absolute rates in hospitalized or non-hospitalized COVID-19 groups that were not significantly higher than those in the control group without infection. The thicker horizontal bars represent the excess rates in the hospitalized COVID-19 group compared to the non-hospitalized COVID-19 group, where the red bars indicate significantly different rates and gray bars indicate no statistical difference. The thinner horizontal bars closer to the *y* axis represent the absolute rate in the non-hospitalized COVID-19 group compared to the control group without infection. The organ systems in **b**, **c**, **d** and **e** were sorted based on the statistical significance and magnitude of differences between hospitalized and non-hospitalized COVID-19 groups (the horizontal red/gray bars between two dots).

Compared to those who were not hospitalized, people who were hospitalized during the acute phase of SARS-CoV-2 infection had significantly higher risk and burden of overall PASC and sequelae in every organ system at each timepoint (Fig. 6 and Supplementary Table 6) and longer risk horizons (Fig. 6 and Supplementary Table 6). The cumulative DALYs of the hospitalized people at 3 years (766.2 per 1,000 persons; 95% CI: 731.7–803.3) were 8.4 times higher than the non-hospitalized group (91.2 per 1,000 persons; 81.6–101.0) (Fig. 6 and Supplementary Table 6).

### Sensitivity analyses

We conducted several sensitivity analyses. (1) We built doubly robust adjustment models in which the covariates were used in both exposure and outcome models, instead of the primary approach where the covariates were applied only in the exposure model. (2) We constructed zero-inflated Poisson models instead of Poisson models in the primary approach. (3) We did not censor participants in the COVID-19 groups upon reinfection, whereas, in the primary approach, participants in the COVID-19 groups were censored upon reinfection. (4) We additionally adjusted for 100 algorithmically selected high-dimensional covariates, instead of only using a set of pre-specified covariates in our primary approach. (5) Instead of defining hospitalization during the acute phase as inpatient admission date within 7 d before or within 30 d after the positive test in the main analyses, we used an alternative definition of hospitalization as inpatient admission date on the day of the positive test or within 30 d after the positive test. (6) We truncated propensity score weights at 99.5% percentiles rather than the 99.9% percentiles in the main analyses. (7) We estimated the IRRs among a sample with complete data on all covariates (*n* = 4,432,414, 83.0% of the full sample) to test the consistency of the results with those obtained using multiple imputation for missing data. (8) We estimated the risks based on Fine–Gray models where death and SARS-CoV-2 infection during follow-up were considered as competing risks. (9) We applied inverse probability of censoring weight to account for non-random censoring due to death or SARS-CoV-2 infection during follow-up across the three groups (the control group without infection, the non-hospitalized COVID-19 group and the hospitalized COVID-19 group). (10) We alternatively used a narrower definition of PASC that included 73 outcomes instead of the 80 outcomes included in the primary analyses. The results from these sensitivity analyses are consistent with those from the main analyses (Supplementary Table 7).

### Negative outcome control analyses

We examined the association between COVID-19 and incident neoplasm as a negative outcome control. The results suggested neutral associations between COVID-19 and the negative outcome control in non-hospitalized participants (IRRs of 1.03 (0.98–1.08) in the first year, 0.94 (0.87–1.01) in the second year and 0.95 (0.88–1.03) in the third year) and hospitalized patients (IRRs of 0.93 (0.82–1.05) in the first year, 0.92 (0.72–1.12) in the second year and 0.93 (0.68–1.08) in the third year).

## Discussion

In this study of 135,161 people with SARS-CoV-2 infection and 5,206,835 controls followed for 3 years, we show that, among non-hospitalized individuals, the increased risk of death was no longer present after the first year of infection, and DALYs and risks for post-acute sequelae declined substantially over the 3 years, with 41.2 sequelae per 1,000 persons and 9.6 DALYs per 1,000 persons emanating from the third year. Among hospitalized patients, the risk of death declined over the 3 years but remained significantly elevated in the third year after infection (29% increased risk and excess burden of death of 8.16 per 1,000 persons). Risks for post-acute sequelae declined over the years, but substantial residual risk remained in the third year, leading to 252.8 sequelae per 1,000 persons and 90.0 DALYs per 1,000 persons. In aggregate, our findings show reduction of risks over 3 years of follow-up but persistent increased risks of major adverse outcomes among hospitalized individuals.

This 3-year follow-up study extends findings from our previous 2-year follow-up analysis^20^. At 2 years, PASC contributed about 81 and 676 DALYs per 1,000 persons in non-hospitalized and hospitalized individuals, respectively. In the third year of follow-up, PASC additionally yielded 9.6 and 90.0 DALYs per 1,000 persons in non-hospitalized and hospitalized individuals, respectively. These results are aligned with the single 3-year study of PASC that has been published to date showing persistent symptomatology in these organ systems^21^ and the contributions of Taquet et al.^15^ showing variation in risk horizon for neuropsychiatric sequelae at 2 years. The epidemiologic evidence of risk persistence provided here is also aligned with studies by Peluso et al.^22^ in which they show that SARS-CoV-2 infection may result in persistent T cell activation in a variety of body tissues that may still be evident 2–3 years after initial mild infection and may associate with PASC, suggesting that even remote and clinically mild SARS-CoV-2 infection could have long-term consequences on tissue-based immune homeostasis.

Lessons from post-acute infection syndromes suggest two key observations: (1) risks for many conditions in the post-acute phase abate with time (that is, risk becomes equal in the infected and control groups a few months to a few years after initial infection) and (2) latent effects may exist, as exemplified by Epstein–Barr virus leading to multiple sclerosis or polio virus leading to post-polio syndrome many years or decades after the initial infection^23–25^. Because SARS-CoV-2 is a novel virus (that was detected only in late 2019), follow-up of infected individuals is only available for a few years. At 3-year follow-up, there was no evidence in our cohort for new adverse outcomes that were not previously manifest. Longer-term studies with careful follow-up of infected individuals to assess the risk trajectories are critical to better understand the risk horizon of health effects and to detect putative latent effects^15^.

Prior epidemiologic characterization of PASC reported the increased risk according to severity of infection^26^; our analyses further add that not only the risk is higher among hospitalized versus non-hospitalized groups at each timepoint but also that the risk persists longer among hospitalized patients, compounding their cumulative burden of health loss. Whereas the 3-year cumulative burden of DALYs due to PASC was high among the non-hospitalized group (91.2 DALYs), it was nearly 8.4 times higher among the hospitalized group (766.2 DALYs), reflecting the sizable toll of health loss in this group. The mechanisms of the longer risk persistence in people with more severe acute disease are not entirely clear. The explanation may be related, in part, to the vulnerability of people who develop severe COVID-19 with respect to more co-existing medical conditions, immune system dysfunction or genetic predisposition^27–31^. Studies have reported that, in severe COVID-19, SARS-CoV-2 leads to systemic infection with viral replication in extrapulmonary sites and RNA persistence in various tissues, including the organ systems that display persistent risks in our analyses^32,33^. Whether and to what extent injury during the acute phase and/or viral persistence plays a mechanistic role in the prolonged risk horizon in these organs is not clear. Both the higher-risk and longer-risk horizon of post-acute sequelae and the persistently increased risk of death at 3 years among those hospitalized for COVID-19 compared to the non-hospitalized COVID-19 group suggest that severity of acute infection is a key driver of the expression of long-term adverse health outcomes. Reducing the risk of hospitalization (by increasing uptake of vaccination and antivirals), prevention of development of sequelae in those who are hospitalized and early recognition and care of PASC in impacted individuals are key strategies to reduce risk of chronic health loss in people with SARS-CoV-2 infection.

Although we emphasize the high toll health loss due to PASC in people who develop severe COVID-19, and although the risk of PASC (on the relative scale) is smaller in people who had mild COVID-19, their absolute number is much larger (than hospitalized individuals) owing to the much higher proportion of people with mild disease^26^. Consequently, much of the burden of PASC in populations is attributed to mild infection. According to an analysis by the Global Burden of Disease (GBD) collaborators, about 90% of people with PASC had mild COVID-19, suggesting that, although preventing severe disease is important, strategies to reduce the risk of post-acute and long-term health loss in people with mild COVID-19 are also needed^1,26^.

The present study has several strengths. It leveraged the breadth and depth of a nationwide integrated health system in the United States and built a large cohort of 5,341,996 participants and followed them for 3 years. We used advanced statistical approaches to balance baseline characteristics; we estimated the risk of a broad array of sequelae in 10 organ systems in people who were non-hospitalized and hospitalized during the acute phase of the disease; and we provided estimates of risk on both the relative and absolute scale by estimating the number of sequelae in each year and cumulatively at 3 years. We additionally estimated DALYs, which provide a more comprehensive measure of burden that accounts for the occurrence of sequelae and their influence on overall health. We conducted several sensitivity analyses that yielded consistent results; and testing of negative outcome control yielded results consistent with a priori expectations.

This study also has several limitations. The VA population is mostly older, White and male that may not be generalizable to other non-veteran populations. To ensure that participants had 3 years of follow-up data, we enrolled people who had positive SARS-CoV-2 tests in 2020—an era that predates the introduction of COVID-19 vaccines. Consequently, the results here do not represent the long-term health effects of SARS-CoV-2 infection among vaccinated individuals, which, given the effectiveness of vaccines in reducing risk of PASC, may be lower. The risks presented in these results may also not reflect the risks of people with antivirals or those who had SARS-CoV-2 infection with later variants (for example, Omicron). Although we used a large number of pre-defined covariates from multiple data sources, including diagnoses, medications and laboratory test results, and balanced these covariates between the COVID-19 groups and the control group without infection, we cannot completely rule out misclassification bias and residual confounding. We pre-specified a comprehensive set of outcomes that has been reported as sequelae of SARS-CoV-2 infection; sequelae that are yet to be characterized are not included in this analysis. We used a comprehensive definition of PASC encompassing 80 sequelae; narrower or broader definitions may yield different estimates. Burden estimates on the absolute scale also reflect the influence of baseline rates. Because the number of participants varies between the non-hospitalized and hospitalized groups, the 95% CIs should be interpreted in the context of the sample size in each group; similarly, estimates of risks and burdens in years 1, 2 and 3 and cumulatively at 3 years should be interpreted along with their uncertainty intervals. We estimated risks and burdens in years 1, 2, and 3 after SARS-CoV-2 infection, and our estimates reflect the average risk during those time periods. Although the VA COVID-19 data resources comprehensively capture COVID-19 test results from a broad array of data sources (including VA and non-VA data), we cannot rule out the possibility of undiagnosed COVID-19 or COVID-19-positive results that were not captured by the VA data systems. If this occurred in the control group, it may have reduced the estimated burden of PASC. We used the data and methodologies of the GBD study to assign a health burden coefficient for each health outcome^26^. This approach assumes that a health condition has the same health burden coefficient regardless of its cause (for example, COVID-19 or other drivers).

In sum, our findings show substantial reduction of risks of PASC over the 3-year follow up. Small residual risk remains among non-hospitalized individuals, and non-trivial burden of death and health loss is evident among hospitalized individuals.

## Methods

### Ethics statement

This study was approved by the institutional review board of the VA St. Louis Health Care System, which granted a waiver of informed consent (protocol number 1606333).

### Study design and setting

This study was conducted using the electronic health databases of the US Department of Veterans Affairs. The VA operates the largest nationwide integrated healthcare system in the United States, including 1,321 healthcare facilities (172 medical centers and 1,138 outpatient sites) serving more than 9 million US veterans each year. Veterans enrolled in the VA healthcare system have access to a comprehensive array of medical services, including outpatient care, inpatient care, prescriptions, mental care, home healthcare, primary care, specialty care, geriatric and extended care, rehabilitation services, medical equipment and prosthetics.

### Data sources

The electronic medical databases of the VA house comprehensive information on outpatient and inpatient encounters, laboratory test results and medications during routine healthcare encounters and are updated daily^25^. Data sources also included the VA Beneficiary Identification Record Locator System, the Medicare Vital Status File, the Social Security Administration Master File and the National Cemetery Administration. We used the inpatient and outpatient domains of the VA Corporate Data Warehouse databases to obtain information on diagnoses and procedures^34–36^. The outpatient pharmacy and barcode medication administration domains were used to obtain data on pharmacy records. The laboratory results domain was used to obtain data on laboratory measurements. Test results on SARS-CoV-2 infection were obtained from VA COVID-19 Shared Data Resource, which consisted of results from polymerase chain reaction tests, antigen tests conducted in the VA or tests reported to the VA^37^. Medicare inpatient and outpatient data were from the VA Centers for Medicare and Medicaid Services (CMS). The area deprivation index (ADI) was used as a summary metric of contextual socioeconomic disadvantage (income, education, employment and housing quality)^38^.

### Cohort

**Extended Data Fig. 5:**
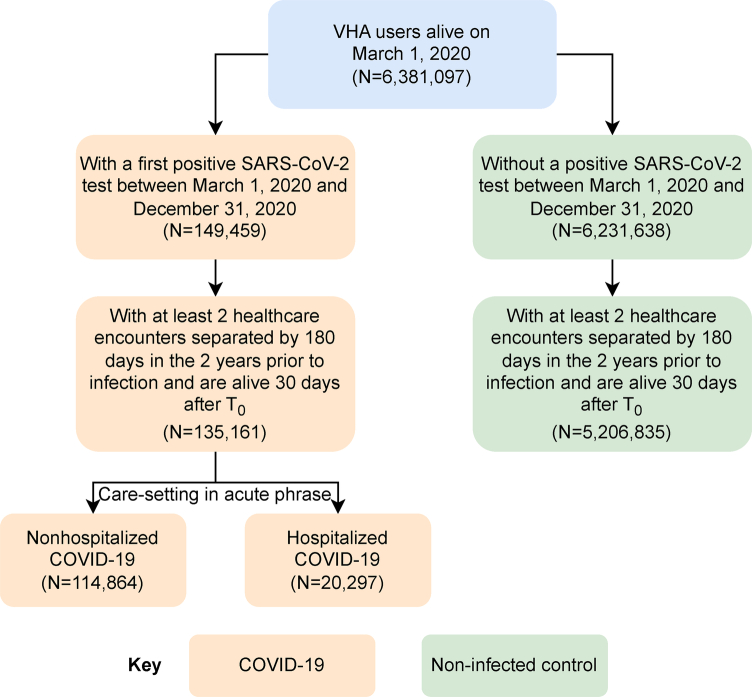
**Cohort flowchart**.

A flowchart showing the cohort construction is presented in Extended Data Fig. 5. We first identified the exposure group with a first SARS-CoV-2 infection between 1 March and 31 December 2020 (*n* = 149,459). We then selected those individuals who are VA users, defined as having at least two healthcare encounters separated by at least 180 d in the 2 years before the infection (*n* = 143,034). To examine the risk of PASC, we selected individuals who were alive at 30 d after the infection, yielding an analytic cohort of 135,161 individuals in the COVID-19 group. Hospitalization within the acute phase was defined as inpatient admission date within 7 d before or within 30 d after the positive test. The COVID-19 group was then further divided by the care setting during the acute phase of infection into non-hospitalized (*n* = 114,864) and hospitalized (*n* = 20,297) COVID-19 groups. The date of positive SARS-CoV-2 test was defined as *T*_0_, and the follow-up started from 30 d after *T*_0_. Participants were followed until death, repeated SARS-CoV-2 infection, 1,080 d after the first infection or 31 December 2023.

To construct a control group without SARS-CoV-2 infection, we first identified 6,231,638 individuals who were alive on 1 March 2020 and did not have a positive SARS-CoV-2 test between 1 March 2020 and 30 March 2021. We then randomly assigned *T*_0_ for the control group based on the distribution of *T*_0_ in the overall COVID-19 group to ensure that the proportion of participants started at a specific date was the same between the COVID-19 group and the control group; 6,194,973 participants were alive at the randomly assigned *T*_0_. Similar to the COVID-19 group, we also required the control group to have encountered the VA healthcare system on at least two occasions separated by at least 180 d in the 2 years before the assigned *T*_0_, yielding a final analytical cohort of 5,206,835 participants in the control group without infection. The follow-up started from 30 d after *T*_0_, and participants were followed until death, a SARS-CoV-2 infection, 1,080 d after *T*_0_ or 31 December 2023.

### Outcomes

We pre-specified a list of 80 individual outcomes that are well-characterized sequelae of SARS-CoV-2 infection based on previous evidence^1,4–6,8,9,11–13,20,25,37^. These outcomes were defined using International Classification of Diseases 10th Revision (ICD-10) diagnosis codes, medical prescriptions and laboratory measurements^20,25^. Incident outcomes were identified when the outcomes did not occur in the 2 years before *T*_0_ and they were the first occurrences from 30 d after *T*_0_ to the end of follow-up. The individual outcomes were then grouped into 10 organ systems: cardiovascular, coagulation and hematological, fatigue, gastrointestinal, kidney, mental health, metabolic, musculoskeletal, neurological and pulmonary. For overall PASC and outcomes at organ system level, we estimated the number of sequelae as the sum of the occurrence of individual outcomes included in a composite outcome. We additionally used the GBD methodologies to estimate DALYs, which represent a measure of disease burden that accounts for the number of sequelae included and their influence on overall health^20,26,39^. For each individual outcome, a health burden coefficient was assigned^20,25,26^. DALYs were then estimated by the weighted sum of all individual outcomes included in a composite outcome, where weight is the health burden coefficient for each individual outcome^20,25^.

### Covariates

A set of pre-defined covariates was selected based on prior knowledge of potential confounders that may bias the relationship between SARS-CoV-2 infection and PASC^1,4,6–8,12,13,20,25^. The demographic covariates included age, self-reported sex, self-reported race (White, Black and Other), ADI at residential address and smoking status (never, former and current smokers). Additional covariates included estimated glomerular filtration rate (eGFR), systolic and diastolic blood pressure and body mass index measured before and closest to *T*_0_. A set of variables defining healthcare utilization included the use of long-term care in the year before the pandemic, receipt of seasonal influenza vaccination each year for up to 5 years before *T*_0_, the number of inpatient and outpatient Medicare visits in the year before the pandemic, the number of inpatient and outpatient unique medical prescriptions and and the number of inpatient and outpatient laboratory panels in the VA medical system separated by 180-d intervals. Comorbidities included anxiety, cardiovascular disease, cerebrovascular disease, chronic kidney disease, chronic lung disease, dementia, depression, diabetes, immunocompromised status (history of organ transplantation, end stage kidney disease, cancer, HIV or prescriptions of corticosteroids or immunosuppressants) and peripheral artery diseases. To account for spatiotemporal variations, we accounted for the calendar week of SARS-CoV-2 infection for the COVID-19 groups or the assigned *T*_0_ for the control group as well as the geographic location of medical service. Missing values included 9.2% for eGFR, 4.9% for systolic and diastolic blood pressure and 10.4% for body mass index. We imputed the missing data using multivariate imputation by chained equations and matching method with predictive mean conditional on all covariates in COVID-19 groups and the control group separately^25^. All the covariates were measured using a look-back period of 2 years before *T*_0_ unless otherwise specified.

### Statistical analyses

**Extended Data Fig. 6:**
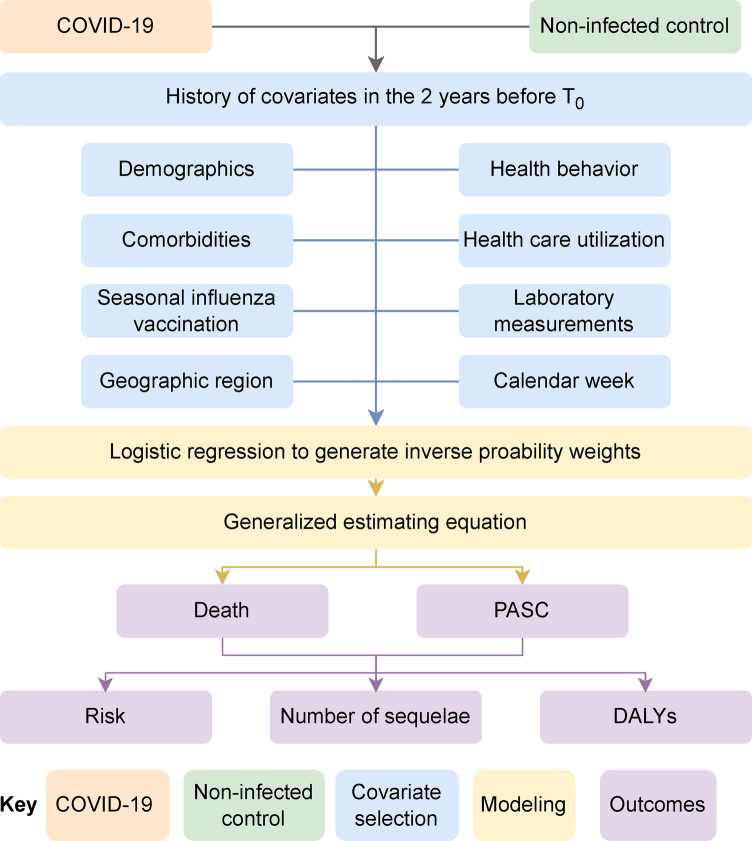
**Analytic flowchart**.

The COVID-19 group was separated by care setting during the acute phase into two mutually exclusive groups: non-hospitalized and hospitalized COVID-19 groups. Baseline characteristics of the COVID-19 groups and the control group without infection were reported. Continuous variables were reported as means (standard deviations), and categorical variables were reported as frequencies (percentages). Standardized mean differences were computed to evaluate covariate balance between COVID-19 groups and the control group without infection, where a value of less than 0.1 was considered evidence of good covariate balance. An analytic flowchart is presented in Extended Data Fig. 6.

Inverse probability weighting was used to balance baseline differences between the two COVID-19 groups and the control group without infection^20^. Logistic regression models were constructed to estimate the probability of being assigned to the target group given the pre-specified covariates (propensity score). To provide a representative risk assessment, we selected the overall population (COVID-19 groups and the control group) as the target population. The inverse probability weights for all three groups were then computed as the propensity score divided by (1 − propensity score). We truncated the propensity score weights at 99.9% percentiles in each group (the control, non-hospitalized COVID-19 and hospitalized COVID-19 groups) to reduce the influence of excessively large weights on the analytical results. We estimated the risk of death and the risk of sequelae at the levels of overall PASC, organ systems and individual outcomes in the weighted cohorts during three time periods: 30–360 d (first year), 361–720 d (second year) and 721–1,080 d (third year) after *T*_0_. To estimate the risk of incident outcome in each period, participants were considered at risk if the examined outcome did not occur in the previous period. We estimated the propensity score weights independently within each period and applied the weights from different periods into one outcome model to estimate the risks and cumulative burden. Participants were censored at the time of death or SARS-CoV-2 infection during follow-up for both COVID-19 groups (non-hospitalized and hospitalized) and the control group.

IRRs, absolute rates, absolute rate differences, cumulative rates, cumulative rate differences for death, the number of sequelae and DALYs were estimated from weighted generalized estimating equations using a log link and a Poisson distribution. The rate differences in death, the number of sequelae and DALYs overall and by organ system between COVID-19 groups and the control group without infection were considered as outcomes due to COVID-19. The percentage contribution of number of sequelae and DALYs in each year during the follow-up were estimated for overall PASC and by organ system. The 95% CIs were generated from the 2.5th and 97.5th percentiles of parametric bootstrapping of 1,000 times based on the point estimates and covariance matrix of the generalized estimating equations^20^. The number of sequelae and DALYs are reported as rates per 1,000 persons.

In all analyses, a 95% CI of IRR that excluded unity or the number of sequelae and DALYs that excluded zero was considered evidence of statistical significance. Analyses were conducted using SAS Enterprise Guide version 8.3 (SAS Institute), and results were visualized using R version 4.3.2.

### Sensitivity analyses

We performed several sensitivity analyses. (1) We estimated doubly robust adjustment models in which the covariates were used in both exposure and outcome models, instead of the primary approach where the covariates were applied only in the exposure model. (2) Instead of the Poisson models in the primary approach, we constructed zero-inflated Poisson models to evaluate how a large number of zero outcomes influences the model fit. (3) Instead of the primary approach where participants were censored at SARS-CoV-2 infection during follow-up, we did not censor participants in the COVID-19 groups upon reinfection and consider reinfection as a natural outcome of the first infection. (4) Instead of using only the pre-defined set of covariates in our primary approach, we additionally adjusted for 100 algorithmically selected high-dimensional covariates^40^. (5) Instead of defining hospitalization during the acute phase as inpatient admission date within 7 d before or within 30 d after the positive test in the main analyses, we used an alternative definition of hospitalization as inpatient admission date on the day of positive test or within 30 d after the positive test. (6) We truncated propensity score weights at 99.5% percentiles rather than the 99.9% percentiles in the main analyses. (7) We estimated the results among a subsample with complete data on all covariates (*n* = 4,432,414, 83.0% of the full sample) to test the consistency of the results with those obtained using multiple imputation for missing data. (8) We estimated the risks based on Fine–Gray models where death and SARS-CoV-2 infection during follow-up were considered as competing risks^41^. (9) We additionally applied inverse probability of censoring weight to account for non-random censoring due to death or SARS-CoV-2 infection during follow-up across the three groups (the control group without infection and the non-hospitalized COVID-19 and hospitalized COVID-19 groups)^42^. (10) We alternatively used a narrower definition of PASC that included 73 outcomes instead of the 80 outcomes included in the primary analyses.

### Negative outcome control

We used the same analytic approach (outlined above) to examine the association between COVID-19 and incident neoplasms as a negative outcome control in each year during the 3 years of follow-up^43^. There is no mechanistic support for or clinical evidence of a causal relationship between SARS-CoV-2 infection and the risk of incident neoplasms. Reproducing the a priori expected null association between COVID-19 and the negative outcome control may reduce concerns about possible spurious biases^43^.

### Reporting summary

Further information on research design is available in the Nature Portfolio Reporting Summary linked to this article.

## Online content

Any methods, additional references, Nature Portfolio reporting summaries, source data, extended data, supplementary information, acknowledgements, peer review information; details of author contributions and competing interests; and statements of data and code availability are available at 10.1038/s41591-024-02987-8.

### Supplementary information

Reporting Summary

Supplementary Tables **Supplementary Table 1**. Demographic and health characteristics of individuals with COVID-19 separated by care setting of the acute phase and the control group without infection before weighting. **Supplementary Table 2**. Demographic and health characteristics of individuals with COVID-19 separated by care setting of the acute phase and the control group without infection after weighting. **Supplementary Table 3**. IRR, number of sequelae and DALYs due to COVID-19 in the non-hospitalized COVID-19 group, number of sequelae and DALYs in the non-hospitalized COVID-19 group and the control group without infection in 3 years after SARS-CoV-2 infection. **Supplementary Table 4**. IRRs and 95% CIs for 80 individual outcomes in the non-hospitalized and hospitalized COVID-19 groups compared to the control group without infection in 3 years after SARS-CoV-2 infection. **Supplementary Table 5**. IRRs, number of sequelae and DALYs due to COVID-19 in the hospitalized COVID-19 group, number of sequelae and DALYs in the hospitalized COVID-19 group and the control group without infection in 3 years after SARS-CoV-2 infection. **Supplementary Table 6**. Differences in number of sequelae and DALYs between the hospitalized and non-hospitalized COVID-19 groups in 3 years after SARS-CoV-2 infection. **Supplementary Table 7**. Sensitivity analyses and negative outcome control analyses.

## Data Availability

The data that support the findings of this study are available from the US Department of Veterans Affairs. VA data are made freely available to researchers behind the VA firewall with an approved VA study protocol. For more information, visit https://www.virec.research.va.gov or contact the VA Information Resource Center at VIReC@va.gov.

## Code availability

The analytic code is available at https://github.com/caimiao0714/COVID3year.
